# Input-Dependent Wave Attenuation in a Critically-Balanced Model of Cortex

**DOI:** 10.1371/journal.pone.0041419

**Published:** 2012-07-25

**Authors:** Xiao-Hu Yan, Marcelo O. Magnasco

**Affiliations:** Laboratory of Mathematical Physics, Rockefeller University, New York, New York, United States of America; National Research & Technology Council, Argentina

## Abstract

A number of studies have suggested that many properties of brain activity can be understood in terms of critical systems. However it is still not known how the long-range susceptibilities characteristic of criticality arise in the living brain from its local connectivity structures. Here we prove that a dynamically critically-poised model of cortex acquires an infinitely-long ranged susceptibility in the absence of input. When an input is presented, the susceptibility attenuates exponentially as a function of distance, with an increasing spatial attenuation constant (i.e., decreasing range) the larger the input. This is in direct agreement with recent results that show that waves of local field potential activity evoked by single spikes in primary visual cortex of cat and macaque attenuate with a characteristic length that also increases with decreasing contrast of the visual stimulus. A susceptibility that changes spatial range with input strength can be thought to implement an input-dependent spatial integration: when the input is large, no additional evidence is needed in addition to the local input; when the input is weak, evidence needs to be integrated over a larger spatial domain to achieve a decision. Such input-strength-dependent strategies have been demonstrated in visual processing. Our results suggest that input-strength dependent spatial integration may be a natural feature of a critically-balanced cortical network.

## Introduction

The brain analyses sensory input under a wide range of input regimes, and employs a wide variety of strategies to extract information in diverse and challenging environments. A number of strategies employed in the primary sensors, such as gain control [1] or light-level-dependent receptive field shapes [2], have been intensively studied and shown to be optimal computational strategies to make the best use of the signal-to-noise available [3]. Less is understood at the level of the primary sensory cortices, such as strategies that change the scale of spatial integration according to input strength [4]–[6]. A recent study has shown [7] that effective lateral interactions in the primary visual cortex of cats and monkey appear to be modulated by the contrast level in the input. At high stimulus contrasts, small amount of lateral interactions are measured. As the contrast is lowered, waves propagate further away from their place of origin, until at zero contrast spontaneous waves of activity appear to decay extremely slowly with distance to their epicenter. This behavior has been interpreted as gating of the functional connectivity by the input.

We offer a simple model which quantitatively reproduces this phenomenology with minimal assumptions. The central hypothesis is that interactions between neurons are adjusted in such a way that excitation and inhibition are precisely balanced, not just globally, but in local patches. By construction our model is poised at a massive, high-dimensional Hopf bifurcation. A recent model of self-tuned critical neural nets has shown how anti-Hebbian interactions lead to such dynamical states and maintain them, and explained their relationship to self-organized criticality [8]; in our construction, we shall forgo the dynamical poising mechanisms and assume the system is already poised at such a state, to analyze the dynamical consequences of such poising. The second postulate is that interactions are local within the two-dimensional surface of our abstract cortex, and that nonlinearities are local, in fact confined to individual neurons. From these postulates we shall show it follows that the system possesses infinitely-long-ranged susceptibilities at zero input, and that the range of the susceptibility, measured by its exponential decay length, decreases with increasing input.

The ease with which the brain changes tasks and activities, adapts to new sensory regimes or environments, and with which different areas communicate has long suggested to researchers that some features of brain function, and in fact of many biological systems, can be fruitfully studied in the context of critical systems [9]. Such systems have hitherto come in two varieties. *Statistical criticality* refers to systems related to statistical-mechanics models of phase transitions at a critical point [10], [11]; the system may require tuning of an external parameter [12] (a temperature or magnetic field in the original models), or may be *self-organized*, meaning the system spontaneously operates in a regime giving rise to wide (usually power-law) distributions, such as avalanche behavior 13–15 and long-range susceptibilities [16], [17].

A related but different concept, based on the notion of a dynamical bifurcation, is *dynamical criticality*, broadly inspired in the classic works of Gold [18] and Ashby [19]. These systems are poised at the onset of dynamical instabilities such as a Hopf bifurcation [20]–[23], or create an otherwise marginal configuration such as a line attractor [24]–[26]. It again comes in two varieties, one in which a parameter has to be adjusted to achieve poising, and *self-tuned criticality* [21], in which the system spontaneously poises itself at the dynamical transitions [27] Dynamical criticality shows a number of the hallmarks of statistical criticality, including avalanche-like bursts of activity [8]. We show the presence of another hallmark: an infinitely long-ranged susceptibility. But this susceptibility does not take an arbitrary form; as we shall show, this susceptibility emerges in the absence of an input to the system, while the presence of an input dampens the responses to finite range. This specific signature relates now dynamical criticality also to input-dependent spatial integration.

## Analysis

For a typical differential equation, the characteristic physical scales of its solutions are set by constant parameters that can be read-off directly from the differential equation proper; for example, the decay lengthscale in a production-diffusion-degradation model is the square root of the diffusion coefficient divided by the degradation rate, and is therefore a constant. Even if there was a nonlinear diffusion or nonlinear degradation, the lengthscale would still be the quotient of the diffusion coefficient evaluated at low concentrations over the degradation rate evaluated at low concentrations. However, when an equation is poised at the onset of a dynamical transition, the linearized equation around zero does not fully determine the solution, and higher-order terms need to be employed. As a result, the parameters in question are set by the local values of the equation’s terms evaluated at the solution. For example, consider a system at the onset of a Hopf bifurcation, a model widely employed to describe cochlear dynamics [20], [22], [28]:
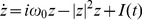
(1)


If a periodic input 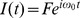 at the resonant frequency 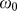 is presented, the solution 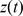 undergoes a transient until it settles into the steady-state solution 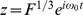.

We now show that the timescale for relaxation of this transient is not set by the coefficients in the equation, but rather by the magnitude of the input. Consider the solution 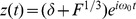, where 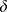 is a distance to the steady-state solution, i.e. the transient proper [29]. Substituting this ansatz into the equation above and canceling terms shows 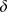 satisfies the equation
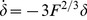
(2)and hence the relaxation timescale is a function of the input amplitude 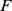 rather than depending only on the internal parameters of the equation. In general, whenever physical scales such as timescales or lengthscales are sensitive functions of the input to the system, and vary with varying input, we may suspect the system to be poised near a dynamical instability conferring on the solution a great deal of sensitivity to the input’s characteristics.

In our abstract model neurons interact with one another through a matrix 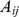 that is assumed to have purely imaginary eigenvalues, and all other interactions are mediated by higher-order terms in the normal form. The equation is
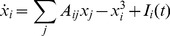
(3)where 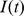 is an input. A fuller treatment would include also an internal noise term 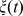 but we shall not use it here.

**Figure 1:**
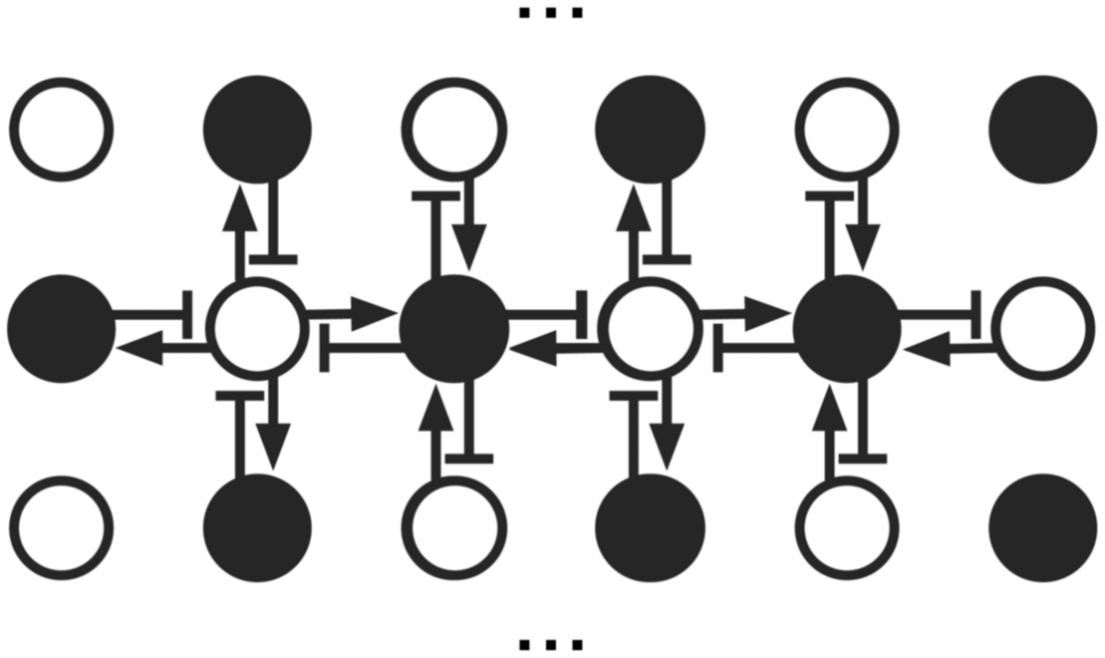
The simplest connectivity of a critically-balanced model of cortex. Black units are inhibitory, white units are excitatory, and all synaptic strengths are identical. Considering a lattice of edge 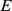, and the site at column 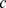 and row 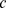 gets index 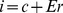, then the matrix is 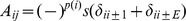 where 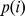 is the parity of site 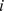; assuming 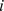 is even, 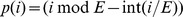.

The simplest such matrix 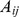 is shown in Figure 1: neurons are arranged on the sites of a chessboard and interact with their 4 nearest neighbors; neurons are declared excitatory if they are on the white squares of the chessboard, inhibitory in the black squares, and the strength of all excitation and inhibition is identical. The interaction matrix 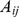 therefore is antisymmetric and has purely imaginary eigenvalues.

It is manifest that equation (3) is a high-dimensional version of equation (1). However care should be exercised in noting that eq (3), unlike (1) is not a normal form for the Hopf bifurcation, because the structure of the nonlinearities in this equation is not generic for a high-dimensional space: the nonlinear terms are local to each of the neurons and do not couple the internal states of different neurons. While we consider this assumption to be reasonable in our context (we do not expect faraway neurons to interact strongly by means other than through their synaptic connections), it is mathematically quite strong.

## Results

We shall now show that equation (3), when equipped with the matrix described in Fig 1, has solutions whose behavior is similar to that experimentally shown in [7] for spike-triggered LFPs in cat and monkey visual cortex.

**Figure 2:**
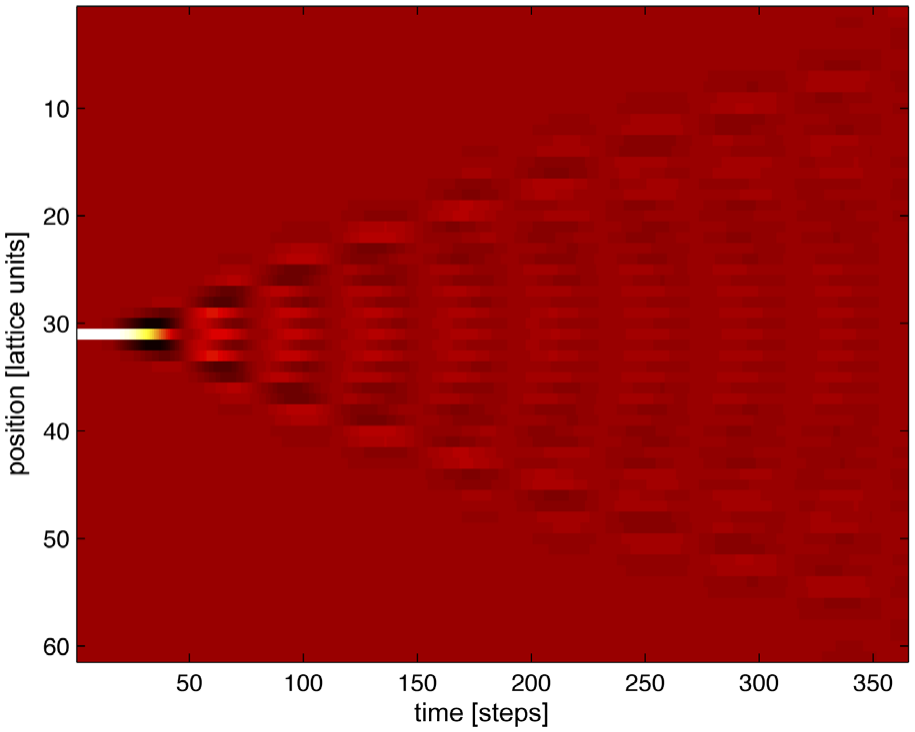
Numerical evolution of equation (1). The activity in a 1D slice of the 2D array (the central row) is displayed. Horizontal axis, time; vertical axis, horizontal coordinate in the array. An initial perturbation at time 0 expands outwards at asymptotically constant speed, with characteristic ripples looking like a “wave” which attenuates algebraically as it goes out.



In the absence of input, eq (3) supports propagating waves which attenuate algebraically, as shown in Figure 2 by direct numerical integration of Eq. (3). This algebraic decay is central to our argument, and important in tying together the dynamically critical models with the more widely studied self-organized critical models, so we shall discuss its origin in a bit more detail. Preserving only the linear part of (3) and iterating it twice to obtain 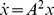, we note the matrix 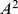 decouples the excitatory and inhibitory sublattices. Concentrating on the excitatory sites, the matrix couples each location in space to the nearby excitatory site in a pattern

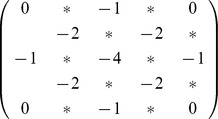
where the 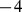 is the self-coupling to the center site. These connections can be seen as a discrete implementation of 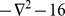, where 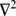 is the Laplacian operator, and suggests we should take a passage to the continuum limit. We shall need to rename our original activities 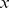 as 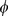, to reuse (in an admitted abuse of notation) the 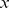 as our spatial variables now, since we need to make space explicit to be able to use 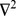. Thus we map 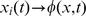 Taking the standard passage to the limit entails taking smaller and smaller lattices, of lattice width 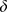, while scaling the original synaptic strength 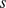 so that the quotient 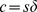 remains finite, and leads to the equation
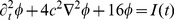
(4)where 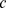 has units of space over time, i.e. a speed. This equation, a Helmholtz equation in euclidean space-time [30], has as a Green function



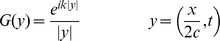
which indeed looks like the propagating waves shown in Figure 2. Care should be taken in referring to these Green functions as waves, because *they are not the solution of a wave equation* –– the equation above is elliptic, not hyperbolic. Pictorially, the equation describes the eigenmodes of a 3D membrane whose coordinates are space (2D) and time (1D). If we look at the solution for one given time, one observes a cut through this shape, which displays peaks and valleys. As one moves forward in time, the valleys and peaks appear to be moving, in what seems to be wavelike fashion. To some extent it is profitable to describe these motions in terms of waves, even though from a strict perspective they are not, because they are not the solution to a wave equation. From an alternative viewpoint we can see the matrix in equation (3), (or alternative the differential operator in equation (5)) has eigenmodes whose spatial frequency 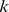 is related to the imaginary component of the corresponding eigenvalue (its temporal frequency 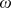) through an equation of the form 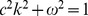, called the *dispersion relation* of the system. Thus 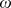 and 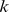 lie on a circle and are inversely related to one another, rather than lying on a line (directly related to one another) which is the dispersion relation of a wave equation. While the system does show a dispersion relation, and to that extent we can describe its behavior as waves, its dispersion relation goes against the normal one for wave equations, with increasing spatial frequency for decreasing temporal frequency.

Returning to the case in which there is a nonzero input, we need to distinguish between the ongoing activity 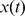 and the extra activity 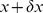 that would be observed elsewhere due to a perturbation at a given site. In the experiment described in [7] the spike-triggered averaging of the LFP is done so as to extract the extra activity that follows a given neural spike at a particular neuron. Since our model is continuous and not formulated in terms of spikes, we shall use a perturbative formulation as the analysis most close in spirit to that experimental measurement. A small perturbation 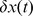 riding on top of a pre-existing activity level 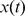 (i.e. a Floquet-Lyapounov analysis [29]) satisfies
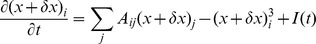
which to lowest order in 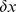 will see the expectation value of 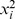 as a damping coefficient (in time):
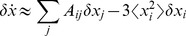
(5)where the 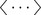 denote an average or expectation value. As the amplitude of stimulation is increased we observe that the waves attenuate exponentially with distance, rather than algebraically, because the variance of the ongoing activity 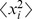 enters the equation for the perturbation as an effective damping coefficient.

Exactly how the variance 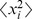 of the response changes with the input’s amplitude will be dependent on the spatiotemporal structure of the input 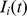; in what follows we shall assume the input to be (or have properties essentially equivalent to) spatiotemporal Gaussian white noise of amplitude 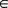:
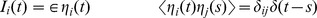
and we will assume that the perturbation 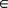 is confined, at time zero, to a single site 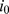: 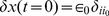. Henceforth we shall describe the wavelike properties of 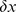, the perturbation riding on top of the ongoing activity 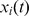 caused by 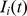


**Figure 3:**
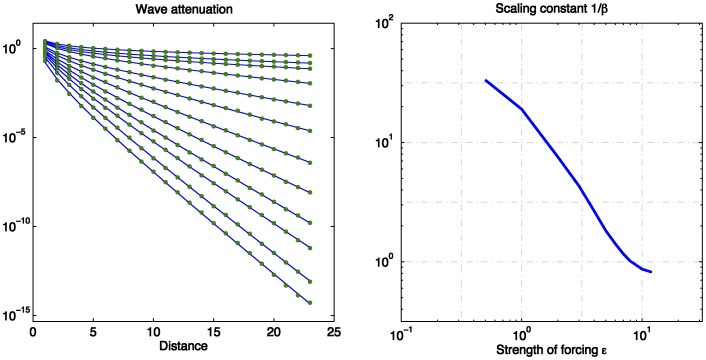
wave attenuation depends directly on the intensity of the input. Left panel, the spatial profile of the wave, for different values of the input intensity; the steeper curves at the bottom correspond to larger intensity, while the shallow and curved solution at the top corresponds to zero input. Right, a fit to the slope of the rightmost points of the left panel, plotted as 1/slope (i.e. attenuation length) as a function of the forcing strength.



At short times 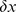 initially spreads just like in Figure 2. As the “wave” propagates outwards at constant speed, the temporal damping of 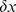 in equation (4) manifests as a spatial damping, so that at large distances the “wave” is exponentially attenuated with distance because it takes a time 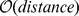 to reach there. This effect is shown in Figure 3, where numerical solutions are shown of the perturbation 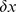 riding on top of a solution 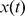 in response to input of specified amplitude. More specifically, Figure 3a shows the maximum value 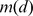 that 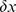 acquires at any given time for times 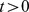, for a given specified distance 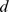 from the epicenter of the perturbation as a function of distance 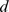:

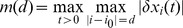
and Figure 3b shows the range 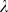, the reciprocal of the decay constant 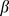 of 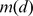, as a function of the input’s strength 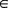:



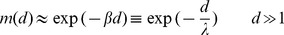



## Discussion

Recent work [7] addressed an ancient dispute in visual neurophysiology: whether visual cortex is primarily driven by its inputs, or whether it is primarily driven by lateral recurrent interactions within cortex, with a relatively small nudge from the inputs. In support of the first view, substantial work from many labs had shown that the responses to visual stimulation in cortex do not change substantially if cortex is inactivated––if it is inhibited from firing through pharmacological or electromagnetic means. Supporting the second view, similarly substantial work from many labs had shown that the strength of intracortical inhibition and excitation is much larger than the overall input from thalamus––it is the nearly perfect balance between excitation and inhibition which permits thalamus to sway activity one way or the other; in fact, in the absence of stimulation cortex activates spontaneously in patterns which agree with those that would have been evoked by pieces of imagery––it literally hallucinates little lines [31]. The results in [7] show that both views are correct at different strengths of the driving input, in this case the visual contrast: at high contrast there is little lateral interaction, while at low or null contrast lateral interactions dominate.

We have demonstrated an extremely compact model reproducing the results of [7]. Our model has but one strong assumption: precise balancing of excitation and inhibition. It offers over the horizontal connection gating model three advantages. First, there is no gating of connectivity at all in our model, as the model has constant parameters without nonlinear interactions; all nonlinearities are strictly local. Gating could be tricky to implement because it cannot be a function of the output of cortex, but rather only of its input, requiring thus that thalamic input to cortex be duplicated: one copy to drive the cortical neurons, the other copy to gate the lateral connections between neurons. Second, the attenuation length is naturally a graded function of the magnitude of the input. In a gated connectivity model it would be extremely hard to arrange the gating to be such a graded function over a wide range of input contrasts. Third, the attenuation length, in the absence of input, is naturally infinite because the balanced cortex produces algebraically-decaying waves. In a functional gating scenario, signal propagation in the absence of input still could either grow or shrink exponentially; to have an algebraic propagation already presupposes balancing internal parameters precisely, which is mostly our only assumption.

Future work on this model should address whether the predicted propagation speed of the outward wave matches the physiologically observed 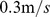, even when taking into account physiologically-relevant synaptic delays. Understanding this will require further elucidation of whether such waves “take shortcuts” through long-range connections, and what their effective speed is when propagating through a tissue with a large spread in connectivity distances. If the mechanism we propose indeed underlies changing integration scale with input intensity, then further work should also attempt to elucidate functional dependences of the local activity level (and thus the attenuation length) with the input, and relate these predictions, measured in distances on the cortex, to the appropriate sensoritopic scales, such as visuotopic units.
